# A statistical shape modelling framework to extract 3D shape biomarkers from medical imaging data: assessing arch morphology of repaired coarctation of the aorta

**DOI:** 10.1186/s12880-016-0142-z

**Published:** 2016-05-31

**Authors:** Jan L. Bruse, Kristin McLeod, Giovanni Biglino, Hopewell N. Ntsinjana, Claudio Capelli, Tain-Yen Hsia, Maxime Sermesant, Xavier Pennec, Andrew M. Taylor, Silvia Schievano, for the Modeling of Congenital Hearts Alliance (MOCHA) Collaborative Group, Andrew Taylor, Alessandro Giardini, Sachin Khambadkone, Silvia Schievano, Marc de Leval, T. Y. Hsia, Edward Bove, Adam Dorfman, G. Hamilton Baker, Anthony Hlavacek, Francesco Migliavacca, Giancarlo Pennati, Gabriele Dubini, Alison Marsden, Jeffrey Feinstein, Irene Vignon-Clementel, Richard Figliola, John McGregor

**Affiliations:** Centre for Cardiovascular Imaging, University College London, Institute of Cardiovascular Science & Cardiorespiratory Unit, Great Ormond Street Hospital for Children, London, UK; Cardiac Modelling Department, Simula Research Laboratory, Oslo, Norway; Inria Sophia Antipolis-Méditeranée, ASCLEPIOS Project, Sophia Antipolis, France; Bristol Heart Institute, School of Clinical Sciences, University of Bristol, Bristol, UK

**Keywords:** Statistical shape model (SSM), 3D Shape analysis, Coarctation of the aorta, Congenital heart disease, Computational modelling

## Abstract

**Background:**

Medical image analysis in clinical practice is commonly carried out on 2D image data, without fully exploiting the detailed 3D anatomical information that is provided by modern non-invasive medical imaging techniques. In this paper, a statistical shape analysis method is presented, which enables the extraction of 3D anatomical shape features from cardiovascular magnetic resonance (CMR) image data, with no need for manual landmarking. The method was applied to repaired aortic coarctation arches that present complex shapes, with the aim of capturing shape features as biomarkers of potential functional relevance. The method is presented from the user-perspective and is evaluated by comparing results with traditional morphometric measurements.

**Methods:**

Steps required to set up the statistical shape modelling analyses, from pre-processing of the CMR images to parameter setting and strategies to account for size differences and outliers, are described in detail. The anatomical mean shape of 20 aortic arches post-aortic coarctation repair (CoA) was computed based on surface models reconstructed from CMR data. By analysing transformations that deform the mean shape towards each of the individual patient’s anatomy, shape patterns related to differences in body surface area (BSA) and ejection fraction (EF) were extracted. The resulting shape vectors, describing shape features in 3D, were compared with traditionally measured 2D and 3D morphometric parameters.

**Results:**

The computed 3D mean shape was close to population mean values of geometric shape descriptors and visually integrated characteristic shape features associated with our population of CoA shapes. After removing size effects due to differences in body surface area (BSA) between patients, distinct 3D shape features of the aortic arch correlated significantly with EF (*r* = 0.521, *p* = .022) and were well in agreement with trends as shown by traditional shape descriptors.

**Conclusions:**

The suggested method has the potential to discover previously unknown 3D shape biomarkers from medical imaging data. Thus, it could contribute to improving diagnosis and risk stratification in complex cardiac disease.

**Electronic supplementary material:**

The online version of this article (doi:10.1186/s12880-016-0142-z) contains supplementary material, which is available to authorized users.

## Background

Diagnosis and risk stratification of cardiac disease using medical imaging techniques are primarily based on the analysis of anatomy and structure of the heart and vessels. This is particularly true for complex conditions such as congenital heart disease (CHD), where the morphology defines the cardiac defect in the first instance and is subsequently altered by surgical and catheter intervention to improve functionality. In clinical practice, however, anatomical analysis of shape and structure is often carried out via simple morphometry, using parameters measured in 2D (e.g. vessel diameter, area, angulation). This does not fully exploit the abundance of information that current imaging techniques such as cardiovascular magnetic resonance (CMR) or computed tomography (CT) offer [1, 2]. Furthermore, using simple shape descriptors, the relationship between complex global and regional 3D shape features, such as the combination of stenoses, dilations or tortuosity and cardiac function has not been fully explored.

Conversely, statistical shape models (SSM) allow visualisation and analysis of global and regional shape patterns simultaneously and in 3D [3] as they are constituted by a *computational atlas* or *template*, which integrates all anatomical shape information intuitively as a visual and numerical mean shape and its variations in 3D. The template is essentially an anatomical model of the average geometry of a shape population. Based on this template, descriptive or predictive statistical shape models can be built [1, 4], to explore how changes in shape are associated with functional changes.

**Fig. 1:**
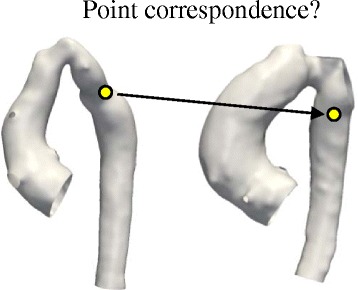
Point-to-point correspondence problem in complex cardiac morphologies. Widely used parametric methods to build statistical shape models are based on the so called Point Distribution Model (PDM) [5], in which shapes are parameterised by landmarks. Bookstein et al. [40] define landmarks as points on the structure’s surface for which “objectively meaningful and reproducible […] counterparts […]” exist in all other structures present in the dataset. In complex cardiac structures however, those point correspondences are difficult to establish, as illustrated here for two aortic arch models from the CoA cohort

SSMs have been applied in cardiac research for around two decades [5] in order to describe 3D morphological characteristics and, more recently, for diagnostic or prognostic purposes [4, 6, 7]. However, these studies are based on parametric methods, in which: i) Shapes are parameterised by landmarks, and ii) Point-to-point correspondence between input shapes is a requirement. These pre-requisites prove particularly challenging to fulfil when dealing with complex, amorphous structures and, therefore, limit the use of such methods in CHD (Fig. 1). In addition, manual landmarking is laborious, limited to expert users [8] and proves to be challenging in the absence of distinct anatomical landmarks.

More recently, a novel *non-parametric* SSM framework that does not rely on any prior landmarking [9, 10], has been introduced to the cardiac field by Mansi et al. [11–13]. The method is based on a complex mathematical framework, which analyses how a representative template shape deforms into each of the shapes present in the population. In a simplified way, for example, an “ideal” template aorta can be deformed into any possible patient aorta shape by applying the correct transformations. Instead of the shapes themselves, these transformations are analysed [14] and subsequent shape analysis is carried out robustly within this transformation framework. A key advantage, in addition to neither requiring landmarking nor point-to-point correspondence between input shapes, is that the method is able to handle large variability between shapes, making it an even more attractive tool for investigating 3D cardiovascular anatomical structures in CHD.

The aim of this paper is to present this shape analysis method to the larger clinical and engineering community by describing a step-by-step approach to set up such a SSM and by demonstrating its validity using conventional morphometric parameters. As an example, the study focuses on aortic arch shapes of patients post coarctation repair [15, 16], as they typically present highly variable, complex shapes, which have been extensively described in terms of traditional morphometric analyses [17–19]. To demonstrate the capabilities of the proposed method, we have derived global and regional shape features potentially associated with ejection fraction (EF) as novel 3D shape biomarkers. We hypothesised that low EF, which characterises poor ventricular function, could be associated with distinct shape patterns of the aortic arch that affect cardiac afterload.

## Methods

### Statistical shape modelling framework (SSM)

**Fig. 2:**
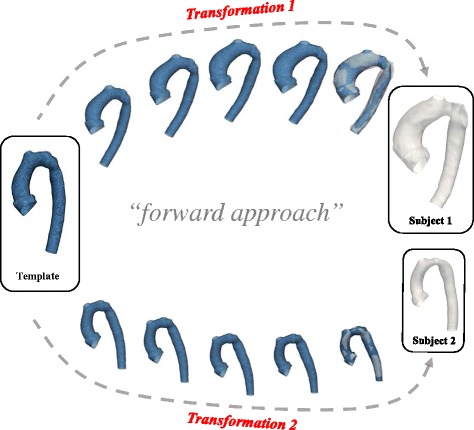
Forward approach: Transformations of the template characterise individual subject shapes. The statistical shape analysis method is based on analysing subject-specific transformations that deform a computed template shape towards each patient shape rather than considering the actual 3D shapes. The transformations are unique for each subject and comprise all relevant shape features that characterise the subject shape

The shape analysis method used here makes use of a framework proposed by Durrleman et al. [12, 14]. To compute a template (i.e. an “anatomical mean shape”) and describe shape variability around this template, the framework is based on a *forward approach* [14], which essentially describes each subject as a deformation of the template plus some residual information (Fig. 2) [12]. The template is deformed into each subject shape by applying an appropriate *transformation*. Thus, the transformation function is the crucial component for shape analysis as it “maps” (i.e. describes how to transform one geometry into the shape of another geometry) the template towards each individual subject shape (Appendix 1).

**Fig. 3:**
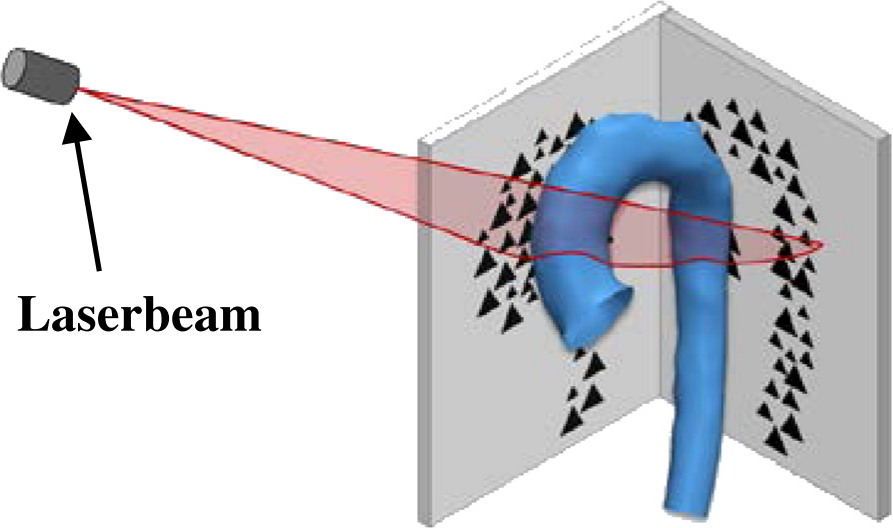
Transferring surface shapes into the space of currents: Analogy to 3D laser scanning of objects. Landmarking of the input shapes is avoided by using *mathematical currents* as non-parametric shape descriptors that model a specific patient shape as a distribution of shape features. Obtaining a currents representation as a surrogate for the actual 3D shape can be compared to probing a surface with a laser beam from different angles and positions

To represent shapes non-parametrically without involving landmarking, the framework relies on *mathematical currents* introduced to anatomical analysis by Glaunès and Vaillant [9]. Currents act as surrogate representations of shapes by characterising a shape as a *distribution of shape features* [14]. Shapes can then be compared by computing how distributions of features differ, rather than by computing differences between individual points. This removes the parameterisation required by other methods. Currents can be seen as an *indirect measure of shape* as they model geometric objects via their reaction on a probing test vector field [20, 21]. An analogy to currents representing shapes could be probing an object with a 3D laser scanner (the “test vector field”) with a certain direction from all possible angles or positions around the object (Fig. 3) [20]. Mathematically, currents are linear applications allowing the computation of the mean, standard deviation, and other descriptive statistics – on 3D shapes.

**Fig. 4:**
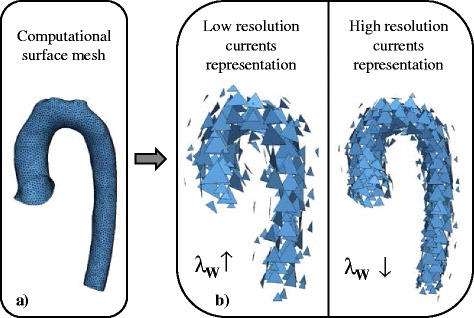
Influence of the resolution parameter λ_W._ One parameter to be set by the user is the currents resolution λ_W_, which controls to which degree shape features of the input 3D shape given as a computational mesh (**a**) are included in the shape’s currents representation. High λ_W_ values neglect small shape features (**b**)

Input shapes are typically given as *computational surface meshes* (Fig. 4a), which provide point coordinates in space and describe how those points are connected. Here, surface meshes define the wall of the aorta, for example. As a first step, the meshes need to be transferred into their currents representation*.* The *resolution* of the currents, λ_W,_ controls to which degree small shape features of the input shapes are included – large λ_W_ result in neglecting small features (Fig. 4b). This becomes particularly useful when it is not desirable to retain small features extracted from the segmentation, which may be caused by image artefacts or suboptimal registration [21]. Once the resolution λ_W_ is set, the template is computed as the average of all currents (Appendix 1).

**Fig. 5:**
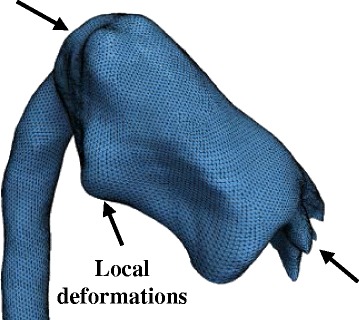
Influence of the stiffness parameter λ_V._ The second parameter to be set by the user, λ_V_, controls the stiffness or elasticity of the deformation of the template towards each subject shape. Low deformation stiffness values result in too local, unrealistic deformations

Unique shape features of each subject are captured by computing the transformations that deform the template towards each subject shape. In order to calculate these transformations, a second parameter λ_V_, which controls the *stiffness* of those deformations, is set: large λ_V_ result in “stiffer” (i.e. less elastic) deformations that capture more global shape features (Fig. 5) [12]. This parameter can be considered as changing the elasticity of the material of the surface models; the more elastic the material, the more the surface models can be manipulated. For example, stretching or deforming a lycra cloth (small λ_V_) will have a different result compared to stretching a leather cloth (large λ_V_).

**Fig. 6:**
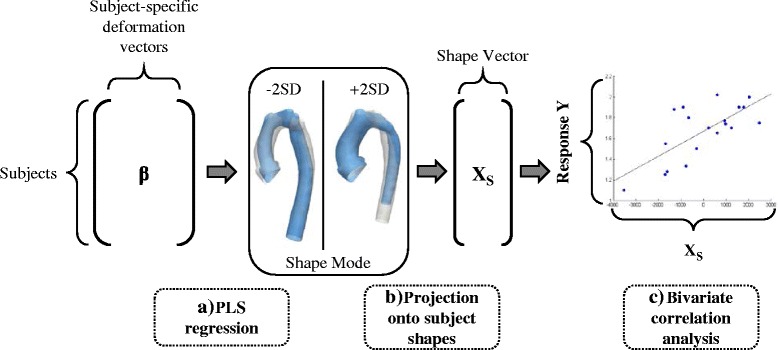
Analysing the output using dimensionality reduction techniques and correlation analyses. PLS regression is used to extract shape patterns most related to a selected response variable as shape modes. Subject-specific deformation vectors, derived from the template computation, constitute the input. Resulting shape modes can be visualised as 3D shape deformations (**a**). By projecting shape modes onto each subject shape, subject-specific shape vectors X_S_ can be derived that constitute a numerical representation of the 3D shape features captured by the shape mode (**b**). X_S_ is correlated with the selected response parameter as measured on the subjects in order to determine how strongly shape and response are associated (c). Analysis techniques are marked with dashed lines

After computing the transformations of the template toward each shape present in the population, each subject shape is uniquely characterised by a multitude of deformation vectors rather than its actual 3D surface. To describe the deformation data with the minimum number of required parameters, a statistical method called partial least square regression (PLS) [12, 22], is employed (Fig. 6a). PLS allows the extraction of *shape modes* [5], which represent the dominant, most common shape features observed in the population that are most correlated with a specific parameter of interest (such as a clinical parameter measured on the individual patient). Here, shape modes most related to body surface area (BSA) and the functional parameter ejection fraction (EF) were extracted.

Extracted shape patterns described by PLS shape modes are visualised by deforming the computed template shape with the transformations along the direction of the mode (Fig. 6a). To determine whether the obtained shape patterns are correlated with a response parameter, shape modes need to be broken down to numbers that allow statistical analysis. This is achieved by mathematically projecting each subject-specific patient transformation onto the found shape mode [12], which yields the so called *shape vector* X_S_ (Fig. 6b). Shape vectors are essentially numerical representations of a specific shape mode. Each shape vector entry describes in one subject-specific number how much the template has to be deformed along the derived shape mode in order to match the specific subject shape as well as possible. The shape vector thus represents 3D global and regional shape features associated with a certain subject and response parameter. Further standard correlation analysis between the shape vector and the response parameter reveals how well subject shape features are represented by the derived shape mode (Fig. 6c). A perfect correlation of shape vector and response would imply that the derived shape mode showed exactly those shape patterns associated with low or high response values (such as high or low EF) when moving along the shape mode from low to high shape vector values.

For mathematical details about the shape modelling process as outlined above, we refer to Appendix 1 and the referenced literature. The entire mathematical framework has been published under the name “exoshape” and is publicly available as a Matlab (The MathWorks, Natick, MA) code [12, 22], (https://team.inria.fr/asclepios/software/exoshape/). A similar, open-source code has been recently published in C as “Deformetrica” by Durrleman et al. [23] (http://www.deformetrica.org/).

**Fig. 7:**
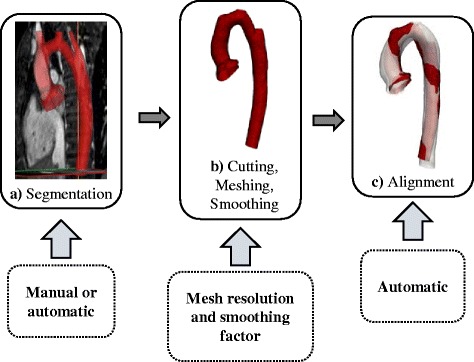
Overview of pre-processing steps prior to shape analysis. Cardiac structures of interest are segmented manually or automatically from 3D imaging data (**a**). Segmented models then are cut, appropriately meshed and smoothed in order to remove irrelevant shape variability (**b**). Before running the shape analysis, the resulting surface models are aligned i.e. rigidly registered in order to reduce bias due to differences in scaling, transformation and rotation (**c**). User interaction is marked with dashed lines

The described SSM framework was applied to the CoA patients following the steps as explained in detail in the next sections (Fig. 7): i) Segmentation of patient CMR images to reconstruct the 3D surface models of the structures of interest; the models and CMR data were also used to compute traditional 2D or 3D morphometric parameters (Fig. 7a); ii) meshing and smoothing of the segmented models to create the computational input for the template calculation (Fig. 7b); iii) registration of the input shapes; (Fig. 7c) and iv) setting of resolution λ_W_ and stiffness λ_V,_ which are the crucial parameters the user needs to provide along with the input shapes prior to calculating the template.

After the template is computed, the following post-processing analyses are carried out: i) removing confounders such as size differences between subjects prior to extracting shape features related to the functional parameter EF as they can hide potentially important shape features; ii) accounting for outliers and influential subjects that are common in clinical data of pathological shapes; iii) validating the template as representing the mean shape of the population and as being not substantially affected if any of the shapes that were used to compute it is removed or if a new patient is added iv) analysing associations between extracted shape features (represented by shape vectors as well as by traditional 2D and 3D measured geometric parameters) and demographic (BSA) and functionally relevant parameters (EF) via standard bivariate correlation analysis (Fig. 6c).

### Patient population, image data and 2D morphometry

CMR imaging data from 20 CoA patients post-repair (16.5 ± 3.1 years, range 11.1 to 20.1 years; CoA repair performed at 4 days to 5 years of age) were included in the study. Conventional morphological descriptors for this population were previously reported by our group [24].

Three-dimensional volumes of the left ventricle (LV) and the aorta during mid-diastolic rest period were obtained from CMR using a 1.5 T Avanto MR scanner (Siemens Medical Solutions, Erlangen, Germany) with a 3D balanced, steady-state free precession (bSSFP), whole-heart, free breathing isotropic data acquisition method (iso-volumetric voxel size 1.5 × 1.5 × 1.5 mm) [24]. Ejection fraction (EF) was measured from the CMR data [24].

Images were segmented using thresholding and region-growing techniques combined with manual editing in commercial software (Mimics, Leuven, Belgium) [24]. A previous study comparing physical objects and their respective 3D segmented and reconstructed computer models found an average operator induced error in the order of 0.75 mm, which equals about half the voxel size in our study [25]. In order to reduce irrelevant shape variability, aortas were cut such that only the root, the arch and the descending aorta up to the diaphragm were kept. As the focus of this analysis lies on the arch shape, coronary arteries and head and neck vessels were cut as close as possible to the arch. This is a common pre-processing step in shape analysis of aortic arches [26–28]. The final segmented surface models of the aortas were stored as computational surface meshes.

**Fig. 8:**
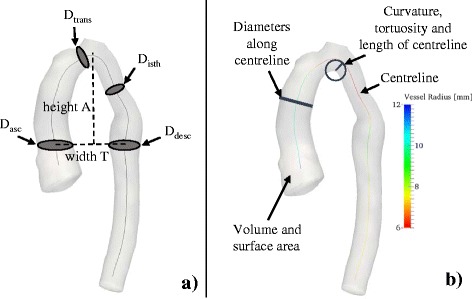
Geometric parameters measured in 2D (**a**) and 3D (**b**). Geometric parameters such as diameters D and aortic arch height A and width T were measured manually on 2D CMR image slices according to [17] and [24] (**a**). 3D parameters were computed semi-automatically using VMTK for all input shapes (**b**)

Conventional 2D morphometry was carried out manually on CMR imaging data to measure the ratio of aortic arch height (A) and width (T) as well as the ascending and descending aortic arch diameters (D_asc_ and D_desc,_ respectively) at the level of the pulmonary artery as proposed by Ou et al. [17] (Fig. 8a). Diameters at the transverse arch level (D_trans_) and at the isthmus level (D_isth_) were measured manually as described previously [24].

3D shape parameters were computed semi-automatically from the segmented arch surface models using The Vascular Modeling Toolkit [29] (VMTK, Orobix, Bergamo, Italy; *www.vmtk.org*) in combination with Matlab. Extracted geometric parameters included volume V and surface area A_surf_, as well as parameters associated with the vessel centreline such as length, curvature and tortuosity [30, 31], and inner vessel diameters along the centreline (minimum D_min_, maximum D_max_ and median diameters D_med_) (Fig. 8b). Table 1 provides an overview of all geometric parameters that were assessed via correlation analyses. Note that all measured geometric parameters were indexed to patient body surface area (BSA), where applicable.

**Table 1:** Morphometric parameters measured on the 3D surface models of the arches

2D measured parameters (manually)	3D measured parameters (VMTK)
• Arch height A [mm] • Arch width T [mm] • Ratio A/T • Diameters at ascending, transverse, isthmus and descending level of the aorta: D_asc_, D_trans_, D_isth_, D_desc_ [mm] • Ratios D_asc_/D_desc_, D_trans_/D_desc_ and D_isth_/D_desc_	• Volume V [mm^3^] • Surface Area A_surf_ [mm^2^] • Ratio A_surf_/V • Centreline length L_CL_ [mm] • Centreline Tortuosity To_CL_ • Median curvature along centreline C_med_ [1/mm] • Maximum, minimum and median diameter along centreline D_max_, D_min_, D_med_ [mm]

### Pre-processing

#### Meshing and smoothing

**Fig. 9:**
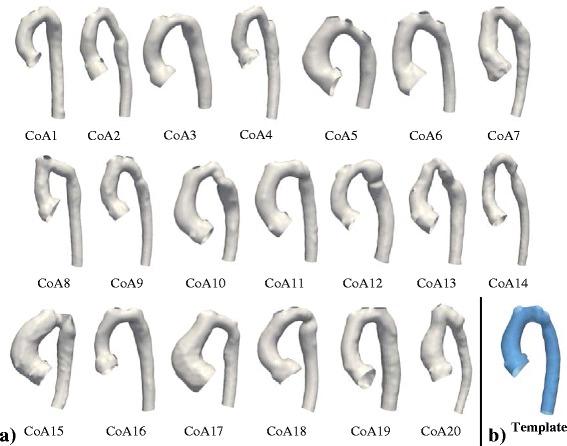
Input surface models of the studied patients post-aortic coarctation repair (**a**) and computed template (**b**). Computational surface meshes of 20 aortic arches constituted the input for the shape analysis (**a**). Coronary arteries and head and neck vessels were removed prior to analysis (3D rotatable models of the arches can be found under *www.ucl.ac.uk/cardiac-engineering/research/library-of-3d-anatomies/congenital_defects/coarctations* *).* The final template (mean shape, blue) computed on the entire population (*N* = 20 subjects) shows characteristic shape features associated with CoA such as a narrowing in the transverse and isthmus arch section as well as a slightly dilated aortic root and an overall slightly gothic and tortuous arch shape (**b**)

Preliminary sensitivity analyses were carried out in order to assess the influence of the meshing, and of the resolution and stiffness parameters (λ_W_, λ_V_) on computational time and on the final template shape (Appendix 2). Results showed that template calculation time can be reduced by up to 85 % if an appropriately low mesh resolution is chosen - without substantially affecting the final template shape. To determine an optimally low, yet sufficient, mesh resolution, we focussed on the smallest subject present in the population of shapes as it defines a lower limit for mesh resolution. Starting from the original surface model of the smallest subject obtained from segmentation (in this case, subject CoA3), re-meshed surface models were created from low (~0.3 cells/mm^2^) to high (~1.5 cells/mm^2^) mesh resolution in VMTK. To quantify deviations from the original segmented shape, the surface area A_surf_ of each re-meshed model was measured and compared to the respective values of the original mesh (A_surf,orig_ = 8825 mm^2^). Surface area deviations were calculated. A cut-off value for tolerable surface errors was chosen to be 0.5 % compared to the original subject mesh, which was reached for a surface mesh resolution of 0.75 cells/mm^2^. All CoA arch surface models were meshed with this resolution, using an additional passband smoothing filter to further reduce unnecessary shape variability (Fig. 9a).

#### Alignment of input meshes

To reduce possible bias introduced by misaligned surface models, a two-step approach is proposed. First, each input shape was aligned (i.e. rigidly registered using translation and rotation only) to an initial reference shape using registration functions based on the iterative closest point (ICP) algorithm available in VMTK [32]. The initial reference shape was determined as the closest shape to the centre or “mean” of the population (in this case subject CoA4; Fig. 9a) according to gross geometric parameters (volume V, surface area A_surf_, centreline length L_CL_ and median diameter along the centreline D_med_). Point-to-point correspondence between the reference mesh and respective subject meshes is not necessary as the correspondence will be updated at each iteration by finding the closest point.

After computing an initial template shape based on the shapes aligned to the initial reference shape (subject CoA4), the final alignment of all input meshes was obtained in a second step by adopting a *Generalised Procrustes Analysis* (GPA) [33] approach in the following manner:

1. The input meshes were re-aligned, with the reference shape this time being the computed template
2. A new template based on the newly aligned meshes was computed
3. The *model compactness* was computed as proposed by Styner et al. [8]
4. If the compactness was decreased, the reference shape was set to the new template and the procedure continued with step 1, otherwise the meshes were aligned sufficiently.

Here, sufficient alignment was obtained after one iteration of the outlined procedure.

#### A priori setting of the resolution and stiffness λ parameters

Generally, it is recommended to set the resolution parameter λ_W_ in the order of magnitude of the shape features to be captured [12]; however, clear indication for parameter setting is missing, in particular for the stiffness λ_V_, which cannot be intuitively estimated. Following sensitivity analysis (Appendix 2), λ_W_ needs to be small enough to be able to capture all the features of interest, while being large enough to discard noise and to allow efficient template computation.

The following approach is proposed to obtain an a priori *estimation* of a suitable set of λ parameters. Essentially, the shape analysis algorithm deforms a template shape towards each individual subject shape present in the population. The quality of the matching of source and target shape depends on the setting of the λ parameters. The suggested approach is based on the idea that the subject with the *most challenging* shape features to be captured defines a lower limit in terms of transformation resolution (λ_W_) and stiffness (λ_V_) to obtain an appropriate matching. Here we assume this to be the smallest subject within our shape population. We therefore transformed an initial template towards the smallest subject shape present in the set of shapes, starting from coarse initial values and decreasing both resolution λ_W_ and stiffness λ_V_ incrementally until a sufficient matching was achieved. Note that incorrectly chosen λ parameters will ultimately result in high matching errors and in unrealistic shape deformations, which can be examined by the user – visually and numerically. To determine starting values for λ_W_ and λ_V_ for computing the initial template, we suggest a “rule of thumb” method, based on the fact that the λ parameters are inherently associated with probing (λ_W_) or deforming surfaces (λ_V_). As both parameters are given as a length in millimetres, they can be squared to define a plane quadratic surface. With this definition, they are interpreted as a percentage of the surface area to be probed or deformed. Based on the smallest surface area A_surf,min_ within the population, λ_W_ and λ_V_ can be initialised using (Eq. 1) for a given percentage p_W_ or p_V_, respectively:

1 $$ {\lambda}_W=\sqrt{p_W\cdot {A}_{surf, min};}{\lambda}_V=\sqrt{p_V\cdot {A}_{surf, min}} $$

For the resolution λ_W_, our approach can be interpreted as probing p_W_ % of the smallest aorta surface area if it was cut open and laid out flat. Note that for large aortas the percentage drops below the chosen percentage values as the same parameters are applied to all shape models. Here, we set p_W_ to 2.5 % and p_V_ to 25 %, which yielded an initial λ_W_ of 15 mm and a λ_V_ of 47 mm, with the minimal surface area present in the set of shapes being A_surf,min_ = 8825 mm^2^. Those values were used to compute an initial template based on all 20 subjects. The initial template was then transformed towards the smallest subject (CoA3) while incrementally decreasing λ_W_ and λ_V_ in 1 mm steps until the matching error between source (initial template) and target (CoA3) was reduced by ≥80 %. A perfect (100 % error reduction) matching is not desired, as for example local shape differences due to segmentation errors or highly localised bulges are not of interest and thus do not need to be modelled. Note that the range of values for λ_V_ was fixed from 47 mm down to 40 mm in order to avoid too local deformations (Fig. 5). Starting from λ_W_ = 15 mm, transformations were computed in parallel for the range of λ_V_ values (47 to 40 mm). If the matching error was not reduced sufficiently by decreasing λ_V_, then λ_W_ was decreased by 1 mm. In this way, we prioritised high λ_W_ values in order to ensure low runtimes for the final template calculation (Appendix 2). The matching error was determined by calculating the maximum surface distances between the target shape (subject CoA3) and the registered deformed source shape (the initial template). Following this procedure, a resolution of λ_W_ = 11 mm and a deformation stiffness of λ_V_ = 44 mm were found to sufficiently reduce the matching error and were used for all further template computations. The template, shape modes and shape vectors were then computed in Matlab based on the 20 arch surface models on a 32GB workstation using 10 cores (runtime for simultaneous template computation and transformation estimation approximately 15 h).

### Post-processing

#### Controlling for confounders and influential observations

Size differences between patients were assumed to be reflected in differences in patient body surface area (BSA). To “normalise” the extraction of functionally relevant shape features, we aimed to remove dominantly size-related shape features first. For that, those shape features most related to a change in BSA were computed using PLS based on the original predictors X_orig_ (the moment vectors deforming the template towards each subject). In previous publications this approach has been used to build a statistical growth model [12, 22]. Here, on the contrary, we aimed to *remove* shape patterns related to size differences between subjects prior to further analyses. A second PLS was then performed on the predictor residuals X_resid_, which were obtained by subtracting the result of the first PLS (the product of PLS predictor scores XS_BSA_ and predictor loadings XL_BSA_) from the original predictors X_orig_ as shown (Eq. 2):

2 $$ {X}_{resid}={X}_{orig}-X{S}_{BSA}\times XL{\mathit{\hbox{'}}}_{BSA} $$

In this way, 3D shape features most related to size differences could be removed prior to analysing correlations of PLS shape vectors with geometric and clinical parameters normalised to BSA [34].

#### Detecting outliers or influential subjects

In preliminary studies, PLS regression proved to be prone to be influenced by outliers. Outliers in terms of shape are common in clinical data of pathological shapes; particularly in the field of CHD, where inter-subject shape variability is typically large. In order to detect influential observations in the PLS regression, the *Cook’s distance* was measured. The Cook’s distance measures how much a specific subject influences the final regression result by leaving out that subject and comparing all remaining fitted values to the original, full data fitted values. It is defined as (Eq. 3) [35]

3 $$ {D}_i=\frac{{\displaystyle {\sum}_{j=1}^n\Big({y}_j-{y_j}_{(i)}}\Big){}^2}{p\cdot MSE};MSE=\frac{1}{n}{\displaystyle \sum_{i=1}^N{\left({y}_i-y\right)}^2} $$

with y_j_ being the j^th^ fitted response variable and y_j (i)_ being the j^th^ fitted response variable if the fit does not include observation i; p is the number of coefficients in the regression model and MSE is the mean square error. The Cook’s distance was computed for each subject by leaving out the subject and performing PLS regression on the remaining subjects. PLS regression was thus repeated N times, with N being the number of subjects. Here, observations with Cook’s distances exceeding four times the mean Cook’s distance were discarded from the analysis as potentially influential observations.

#### Validation of the template - geometric approach

Standard geometric parameters such as Volume V, surface area A_surf_, centreline length L_CL_ and median diameter D_med_ along the centreline of the vessel were computed for each patient shape, averaged over the entire population and compared with the respective parameter measured on the final template shape. The deviations ∆Dev from the mean population values were calculated for x being one of the parameters (V, A_surf_, L_CL_, D_med_) calculated on the template and $\overline{x}$ being the respective population mean as (Eq. 4):

4 $$ \Delta Dev=\frac{x-\overline{x}}{\overline{x}}\cdot 100\% $$

The overall deviation ∆Dev_total_ of the template from population means was calculated as the average of the deviations from each of the above mentioned parameters. A template shape yielding a low overall deviation ∆Dev_total_ from population mean values of below 5 % was considered to represent a good approximation of the mean shape.

#### K-fold cross-validation

In order to assure that the final template shape is not overly influenced by adding or leaving out a specific subject shape, k-fold cross-validation was performed [11]. The entire dataset was divided into k = 10 randomly assigned subsets. The template calculation was run k times, each time leaving out one of the subsets until each patient had been left out once. As the entire set consists of *N* = 20 datasets in total, in each of the k runs N/k = 2 patients were left out. The 10 resulting templates should all be close to the template calculated on the full dataset of *N* = 20 patients. This was assessed visually by overlaying the final template meshes and numerically by measuring the surface distances between each of the 10 templates and the original template.

### Statistical analysis

To back up the findings of the SSM, correlations between the parameters of interest, BSA and EF, with the traditionally measured geometric parameters and demographic parameters (patient age and height) were computed using bivariate correlation analysis. For correlations with BSA, non-indexed geometric shape descriptors were used. In a second step, shape vectors most related to BSA and EF (after removing size effects) were extracted via PLS and were correlated with the response variables BSA, EF, demographic parameters and the 2D and 3D geometric shape descriptors (Table 1). For parameters that were normally distributed, the standard parametric Pearson correlation coefficient r is reported. For non-normally distributed parameters, non-parametric Kendall’s τ is given. Non-normality was assumed if the Shapiro-Wilk test was significant. Parameters were considered significant (2-tailed) for *p*-values < .05. All statistical tests were performed in SPSS (IBM SPSS Statistics v.22, SPSS Inc., Chicago, IL).

## Results

### Computed template and validation

The template shape showed distinct narrowed sections in the transverse arch and isthmus region. The root was slightly dilated and the overall arch shape could be described as rather “gothic” with a narrow arch width T and large height A (Fig. 9b, Additional File 1). Key geometric parameters of the template such as surface area A_surf_, volume V, centreline length L_CL_ and median diameter along the centreline D_med_ were all close to their respective means as measured on the entire population of shapes (Table 2). Overall average deviation from those mean geometric population values was 3.1 %. Thus, the template was considered to be a good representation of the “mean shape” of the CoA population. The cross-validation templates matched the original template well on visual assessment. Using gross geometric parameters (A_surf_, V, L_CL_ and D_med_), cross-validation templates showed average total deviations from the original template ranging from 2.8 to 6.6 %. Average surface distances between the template shapes ranged from 0.21 mm to 1.07 mm. Hence, the computed template was considered to be minimally influenced by adding or removing another patient shape.

**Table 2:** Mean geometric parameters of the population compared to geometric parameters of the template

	Surface Area A_Surf_ [mm^2^]	Volume V [mm^3^]	Centreline Length L_CL_ [mm]	Median Diameter D_med_ [mm]
Mean population values	15392.5	82839.0	224.3	17.1
Template values (λ_W_ = 11 mm, λv = 44 mm)	15351.5	81552.7	215.2	18.2
Deviation from population values	0.3 %	1.5 %	4.1 %	6.4 %
Overall deviation	3.1 %			

λ_W_ and λ_V_ are resolution and stiffness parameters to be set by the user prior to computing the template

### Shape patterns associated with differences in BSA

#### Associations of geometric shape descriptors with changes in BSA

Correlations of the traditionally measured 2D and 3D geometric parameters (Table 1) and demographic parameters with BSA were analysed using non-indexed geometric descriptors. BSA was significantly positively correlated with age (*r* = 0.705; *p* = .001) and height (*r* = 0.838; *p* < .001) and thus accounted for overall size differences between subjects. Further significant positive correlations of BSA were found with volume V (Kendall’s τ = 0.385; *p* = .019) and surface area A_surf_ (*r* = 0.537; *p* = .015) of the arch models, the maximum and minimum diameter along the centreline, D_max_ (τ = 0.460; *p* = .005) and D_min_ (*r* = 0.628; *p* = .003), ascending aortic diameter D_asc_ (*r* = 0.550; *p* = .012), transverse diameter D_trans_ (*r* = 0.453; *p* = .045) and isthmus diameter D_isth_ (*r* = 0.523; *p* = .018) as well as the arch width T (*r* = 0.555; *p* = .011) (Table 3). Significant negative correlations were found with the ratio of arch surface area and volume A_surf_/V (*r* = −0.641; *p* = .002) and the median curvature along the centreline C_med_ (*r* = −0.603; *p* = .005).

**Table 3:** Correlations between BSA and BSA Shape Vector and traditionally measured parameters

Parameter	Body Surface Area	BSA Shape Vector
	BSA [m^2^]	
	*N = 20*	*N = 19*
Body Surface Area BSA [m^2^]	-	*r* = 0.707** (*p* = .001)
Age [years]	*r* = 0.705** (*p* = .001)	*r* = 0.696** (*p* = .001)
Height H [mm]	*r* = 0.838** (*p* < .001)	*r* = 0.872** (*p* < .001)
Volume V [mm^3^]	τ = 0.385* (*p* = .019)	τ = 0.743** (*p* < .001)
Surface Area A_surf_ [mm^2^]	*r* = 0.537* (*p* = .015)	*r* = 0.902** (*p* < .001)
Centreline length L_CL_[mm]	*r* = 0.398 (*p* = .083)	*r* = 0.853** (*p* < .001)
Centreline Tortuosity To_CL_	*r* = 0.022 (*p* = .928)	*r* = 0.206 (*p* = .398)
Ratio A_surf_/V	*r* = −0.641** (*p* = .002)	*r* = −0.787** (*p* < .001)
Median Curvature C_med_ [1/mm]	*r* = −0.603** (*p* = .005)	*r* = −0.718** (*p* = .001)
Maximum Diameter D_max_ [mm]	τ = 0.460** (*p* = .005)	τ = 0.602** (*p* < .001)
Minimum Diameter D_min_ [mm]	*r* = 0.628** (*p* = .003)	*r* = 0.763** (*p* < .001)
Median Diameter D_med_ [mm]	*r* = 0.386 (*p* = .092)	*r* = 0.709** (*p* = .001)
Ascending Diameter D_asc_ [mm]	*r* = 0.550* (*p* = .012)	*r* = 0.708** (*p* = .001)
Transverse Diameter D_trans_ [mm]	*r* = 0.453* (*p* = .045)	*r* = 0.646** (*p* = .003)
Isthmus Diameter D_isth_ [mm]	*r* = 0.523* (*p* = .018)	*r* = 0.746** (*p* < .001)
Descending Diameter D_desc_ [mm]	*r* = 0.332 (*p* = .152)	*r* = 0.740** (*p* < .001)
Ratio D_asc_/D_desc_	*r* = 0.264 (*p* = .260)	*r* = 0.025 (*p* = .918)
Ratio D_isth_/D_trans_	*r* = 0.190 (*p* = .422)	*r* = 0.315 (*p* = .189)
Ratio D_isth_/D_desc_	*r* = 0.364 (*p* = .115)	*r* = 0.278 (*p* = .250)
Ratio D_trans_/D_desc_	*r* = 0.074 (*p* = .757)	*r* = −0.188 (*p* = .442)
Arch height A [mm]	*r* = 0.198 (*p* = .402)	*r* = 0.632** (*p* = .004)
Arch width T [mm]	*r* = 0.555* (*p* = .011)	*r* = 0.626** (*p* = .004)
Ratio A/T	*r* = −0.170 (*p* = .473)	*r* = 0.116 (*p* = .637)

r denotes Pearson’s correlation coefficient for parametric correlations; τ denotes Kendall’s τ for non-parametric correlations; **marks significant level *p* ≤ .001; *marks significant level *p* ≤ .05

#### Associations of shape modes and shape vectors with changes in BSA derived from SSM

A first PLS regression of shape features with BSA revealed subject CoA20 to be influential to the regression as CoA20 exceeded the computed Cook’s distance threshold of 0.77. We considered CoA20 as an outlier in terms of its overall shape as it presented with a highly gothic (A/T ratio = 0.94) arch with a bended descending aorta (Fig. 9a) that is considerably larger than other subjects. Thus, CoA20 is likely to skew the subsequent shape feature extraction and was therefore removed from the following analyses.

**Fig. 10:**
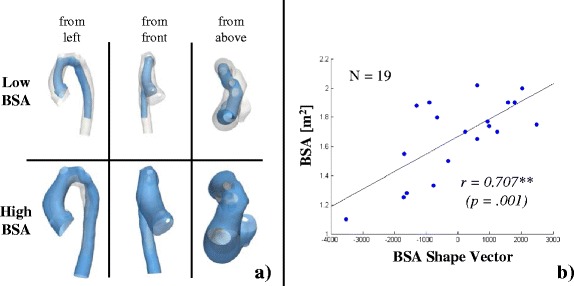
Visualisation of the BSA shape mode (**a**) and correlation with BSA shape vector (**b**). Shape features associated with deforming the template along the BSA Shape Mode from low (a, top) to high BSA values (**a**, *bottom*) from different views as indicated. Low BSA values were associated with a slim, straight and rounded arch shape, whereas moving towards higher BSA values resulted in an overall size increase along with shape deformation towards a more tortuous gothic arch with a slightly dilated root. The measured BSA of the subjects and the shape features as described by the BSA Shape Mode correlated strongly (**b**)

Subsequent PLS regression with BSA on the remaining 19 subjects extracted a BSA shape mode, which accounted for 24 % of the shape variability present in the population. Visually, the BSA shape mode showed an overall enlargement of the deformed template arch shape with an increase in ascending, transverse, isthmus and descending aorta diameter while moving from low towards higher BSA values (Fig. 10a, Additional File 2). The overall arch shape for low BSA was slim and rather straight, with a rounded arch; whereas for high BSA values the arch shape was more gothic and more tortuous with a slightly dilated root and descending aorta.

The correlation between the associated BSA shape vector and BSA was significant with *r* = 0.707 (*p* = .001), implying that the BSA shape mode captured shape features associated with differences in BSA well (Fig. 10b). Furthermore, the computed BSA shape vector correlated positively and significantly with age (*r* = 0.696; *p* = .001) and height (*r* = 0.872; *p* < .001), volume V (τ = 0.743; *p* < .001) and surface area A_surf_ (*r* = 0.902; *p* < .001), centreline length L_CL_ (*r* = 0.853; *p* < .001), diameters D_max_ (*r* = 0.602; *p* < .001), D_min_ (*r* = 0.763; *p* < .001), D_med_ (*r* = 0.709; *p* = .001), D_asc_ (*r* = 0.708; *p* = .001), D_trans_ (*r* = 0.646; *p* = .003), D_isth_ (*r* = 0.746; *p* < .001), D_desc_ (*r* = 0.740; *p* < .001) and arch height A (*r* = 0.632; *p* = .004) and width T (*r* = 0.626; *p* = .004) (Table 3). Significant negative correlations were found for the surface volume ratio A_surf_/V (*r* = −0.787; *p* < .001) and the median curvature C_med_ (*r* = −0.718; *p* = .001). Those associations were correctly depicted by the BSA shape mode (Fig. 10a).

### Shape patterns associated with differences in EF

#### Associations of indexed geometric shape descriptors with changes in EF

Significant positive correlations were found between EF and the ratio of transverse and descending arch diameter D_trans_/D_desc_ (*r* = 0.456; *p* = .050). EF correlated negatively and significantly with the indexed arch surface area iA_surf_ (*r* = −0.571; *p* = .011).

#### Associations of shape modes and shape vectors with changes in EF derived from SSM

**Fig. 11:**
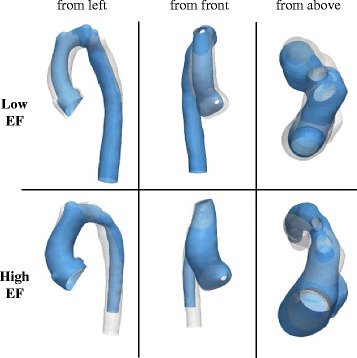
Visualisation of the EF Shape Mode. Shape features associated with deforming the template along the EF Shape Mode from low (*top*) to high EF from different views as indicated. Lower EF was associated with a slim, rather gothic arch shape with a long dilated descending aorta, whereas higher EF was associated with a more rounded arch along with a dilated root and tapering towards a slim descending aorta

A second PLS regression based on the residuals of the first PLS regression with BSA was performed with EF as response. This two-step approach allowed removing shape features due to size differences between subjects prior to extracting shape modes related to EF. This second “normalised” PLS regression yielded the EF shape mode, which accounted for 19 % of the remaining shape variability. The EF shape mode deformed the template from a large, overall rather straight but slightly gothic arch shape with a slim ascending aorta and a dilated descending aorta for low EF values towards a rather compact but rounded arch shape with a dilated aortic root and a slim descending aorta for high EF (Fig. 11, Additional File 3).

**Fig. 12:**
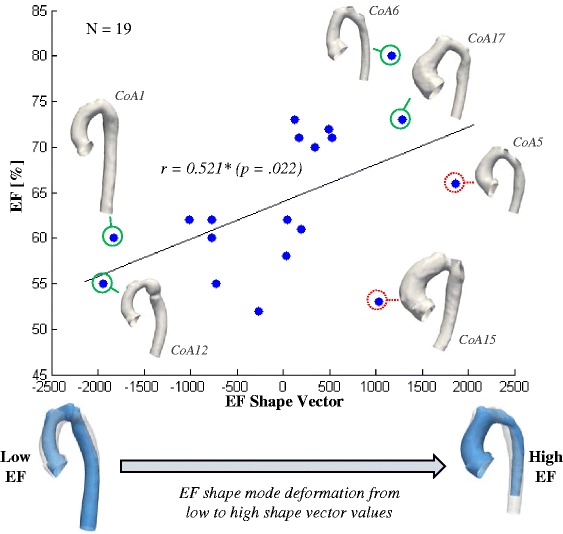
Correlation between EF and EF Shape Vector and visual assessment of results. Measured EF and shape features as described by the EF Shape Mode correlated well. Shape change of the template from a larger arch shape with a slim ascending and a slightly dilated descending aorta was associated with low, negative shape vector values. A smaller arch shape with dilated root and slim descending aorta was associated with high, positive shape vector values (*bottom*). Compared with the shape of two subjects (CoA1 and CoA12) with low EF at the left, lower spectrum of shape vector values, key shape features supposedly associated with low EF values such as a long, slightly dilated descending and a slim ascending aorta, are depicted correctly by the EF shape mode. On the other side of the shape spectrum, subjects CoA6 and CoA17 presented with a high EF and showed shape features in agreement with the shape mode derived for high EF values. Both shapes were compact, with a shorter, slim descending aorta compared to the ascending aorta, along with a dilated aortic root. Two subjects, who most likely contributed to the relatively weak correlation between EF and the EF shape vector, were subjects CoA5 and CoA15 as marked in red (*dashed*). Although they presented with similar shapes as CoA6 and CoA17 and thus do show shape features that should be associated with high EF values, their EF values were in the mid-spectrum for CoA5 and even lower than CoA12 for CoA15

The associated EF shape vector correlated significantly with EF (*r* = 0.521; *p* = .022) (Fig. 12). By analysing correlations of the EF shape vector with measured geometric parameters, further significant positive correlations with the ratio of ascending to descending aorta diameter D_asc_/D_desc_ (*r* = 0.753; *p* < .001) and the ratio of transverse and descending aorta diameter D_trans_/D_desc_ (*r* = 0.457; *p* < .049) were found; corroborating the visual results. Negative significant correlations were found with the indexed descending aorta diameter iD_desc_ (*r* = −0.527; *p* < .020). All further correlations are given in Table 4.

**Table 4:** Correlations between EF and EF Shape Vector and traditionally measured parameters

Parameter	Ejection Fraction EF [%]	EF Shape Vector
	*N* = 19	*N* = 19
Ejection Fraction EF [%]	-	*r* = 0.521* (*p* = .022)
Body Surface Area BSA [m^2^]	*r* = −0.147 (*p* = .548)	*r* = 0.000 (*p* = .999)
Age [years]	*r* = −0.243 (*p* = .316)	*r* = −0.142 (*p* = .561)
Height H [mm]	*r* = −0.391 (*p* = .098)	*r* = −0.246 (*p* = .310)
Volume iV [mm^3^/m^2^]	τ = −0.322 (p = .058)	τ = −0.228 (*p* = .172)
Surface Area iA_surf_ [mm^2^/m^2^]	*r* = −0.571*(*p* = .011)	*r* = −0.320 (*p* = .181)
Centreline length iL_CL_[mm/m^2^]	*r* = −0.255 (*p* = .293)	*r* = −0.338 (*p* = .157)
Centreline Tortuosity iTo_CL_	*r* = −0.039 (*p* = .874)	*r* = 0.267 (*p* = .269)
Ratio A_surf_/V	*r* = −0.386 (*p* = .103)	*r* = 0.072 (*p* = .770)
Median Curvature iC_med_ [1/mm m^2^]	*r* = 0.269 (*p* = .265)	τ = 0.158 (*p* = .345)
Maximum Diameter iD_max_ [mm/m^2^]	τ = −0.096 (*p* = .574)	τ = 0.064 (*p* = .064)
Minimum Diameter iD_min_ [mm/m^2^]	*r* = −0.409 (*p* = .082)	*r* = −0.239 (*p* = .324)
Median Diameter iD_med_ [mm/m^2^]	*r* = −0.363 (*p* = .127)	τ = −0.228 (*p* = .172)
Ascending Diameter iD_asc_ [mm/m^2^]	τ = 0.000 (*p* = 0.999)	τ = 0.170 (*p* = .310)
Transverse Diameter iD_trans_ [mm/m^2^]	*r* = 0.020 (*p* = .937)	*r* = −0.018 (*p* = .942)
Isthmus Diameter iD_isth_ [mm/m^2^]	*r* = −0.407 (*p* = .083)	*r* = −0.441 (*p* = .059)
Descending Diameter iD_desc_ [mm/m^2^]	*r* = −0.442 (*p* = .058)	*r* = −0.527* (*p* = .020)
Ratio D_asc_/D_desc_	*r* = 0.312 (*p* = .193)	*r* = 0.735** (*p* < .001)
Ratio D_isth_/D_trans_	r = −0.362 (*p* = .128)	*r* = −0.453 (*p* = .052)
Ratio D_isth_/D_desc_	*r* = −0.152 (*p* = .535)	*r* = 0.083 (*p* = .736)
Ratio D_trans_/D_desc_	*r* = 0.456* (*p* = .050)	*r* = 0.457* (*p* = .049)
Arch height iA [mm/m^2^]	*r* = −0.154 (*p* = .529)	τ = −0.146 (*p* = .382)
Arch width iT [mm/m^2^]	*r* = 0.000 (*p* = .999)	*r* = 0.018 (*p* = .943)
Ratio A/T	*r* = −0.224 (*p* = .357)	*r* = −0.269 (*p* = .265)

*r* denotes Pearson’s correlation coefficient for parametric correlations; τ denotes Kendall’s τ for non-parametric correlations; **marks significant level *p* ≤ .001; *marks significant level *p* ≤ .05

## Discussion

This study describes and verifies a non-parametric statistical shape analysis method in detail and demonstrates its potential for discovering previously unknown 3D shape biomarkers in a complex anatomical shape population. The methodology is comprehensively explained from the user-perspective, with the aim of making the process more accessible to the broader research community. The shape analysis method was applied to CMR images of the aorta from patients post coarctation repair. The method computes a mean shape for this population of patients – the *template* – that we have shown to have good agreement with the conventional 2D and 3D measurements when averaged across the population (e.g. centreline length of the template = the average of the centreline length measured from each patient). Biomarker information – the shape features – for each individual were then extracted by transforming the mean aorta to each patient’s aorta. These extracted shape features, unique to each individual, were shown to: i) Accurately represent individual characteristics of the arch, as measured by patient-specific 2D/3D morphometric parameters, and ii) Have correlations with body surface area and left ventricular ejection fraction, offering the potential that they may be important biomarkers of biological processes. The found associations of aortic arch shape with ejection fraction were not known previously, which is why we consider the extracted 3D shape features as potential novel shape biomarkers that need to be confirmed by future studies. These results constitute the first statistical shape model of the aorta affected by coarctation.

A description of the statistical shape modelling framework adopted in this study is reported elsewhere in mathematically rather complex terms. Yet, in this paper we present the method from the user’s perspective. Here, we aimed to raise the awareness of the importance of necessary modelling parameters such as the meshing, smoothing and λ parameters for 3D shape analysis of complex anatomical structures. The mesh resolution for the input surfaces mainly affects the computational time needed to compute the template, but does not affect the final template shape substantially. Conversely, the analysis parameters (resolution λ_W_ and stiffness λ_V_) affect both computational time and the final template shape considerably, requiring careful setting according to the shape population to be analysed. We provide tips on how to mesh input models and propose a new way of determining the λ parameters, which ensures robust and efficient template computation, even with an increased number of subjects for future studies. Furthermore, a modified PLS regression technique is described, which enables extraction of shape features independent of size differences between subjects. By measuring the Cook’s Distance during PLS regression, we were able to account for outliers such as one subject with an overly large, “abnormal” aortic shape and indeed a highly impaired cardiac function (EF = 17 %) that had to be excluded in order not to affect the shape feature extraction (subject CoA20). This suggests that the methodology could potentially be used to detect outlying shapes in a complex shape population – which, in turn, might be associated with outlying functional behaviour.

The calculated template based on the 20 CoA cohort showed characteristic shape features associated with CoA such as a slightly gothic arch shape, a dilated root, and a distinct narrowing in the transverse and isthmus arch section. The template shape was validated by comparing its geometry with the population average geometric parameters and by applying cross-validation techniques in order to ensure that removing or adding shapes had no influence. Therefore, new patients can be added easily, which involves performing the described pre-processing steps (segmenting, meshing, cutting, registration) and re-computing the template. Such a template could serve as a representative of the “normal of the abnormal”; a reference mean shape that might facilitate the diagnosis of highly abnormal cases within a pathologic shape population.

Three-dimensional global and regional shape features associated with differences in size (represented by BSA) and function (represented by EF) were extracted and found to be well in agreement with trends confirmed by traditional morphometrics. BSA correlated strongly and significantly with conventional geometric parameters, as expected. Those results confirmed the visual results shown by the SSM, whereby an increase in BSA was associated with an overall increase in aorta length and vessel diameters as well as with a shape development towards a slightly dilated root and a more gothic arch shape. For the first time, high EF was associated with a more compact, rounded arch shape with a slightly dilated aortic root and a slim descending aorta, whereas low EF was associated with a more gothic arch shape, a slim ascending aorta and a slightly dilated descending aorta, which may increase flow resistance across the arch and therefore left ventricular afterload.

Note however, that in order not to inflate Type II error of not detecting actual effects, computed correlation significances were not adjusted for multiple comparisons. Therefore, all results have to be considered as exploratory.

### Analysing the found correlations in detail

#### Correlations with traditionally measured geometric parameters

Whereas BSA correlated strongly with multiple measured 2D and 3D shape descriptors, EF correlated significantly only with two geometrical parameters (the ratio of transverse to descending aortic diameter and the indexed surface area). One reason for this may be that the shape of the aortic arch marginally affects EF. However, these discrepancies could also emphasise that complex 3D shapes cannot always be sufficiently described by traditional individual morphometric measurements. Shape features associated with differences in body size between subjects are typically dominant and contribute to the largest portion of shape variability in natural pathologic shape populations [36]. An increase in body size usually results in an overall size increase of the structure of interest, reflected in increased diameters and vessel length in the case of the aorta. This is why shape features associated with size differences are likely to be picked up by traditional 2D and 3D measurements. For the functional parameter EF though, we were interested in shape features independent of size effects, which, however, may be less prominent and may only be captured by a complex combination and collection of different morphometric parameters. Herein lies the power of 3D statistical shape modelling: results such as the mean shape and its variability are derived as visual, intuitively comprehensible and less biased 3D shape representations taking into account the entire 3D shape, instead of an unhandy collection of multiple measured parameters that might miss out crucial shape features.

#### Correlations with shape vectors describing shape features most related to a specific parameter in 3D

We found a strong significant correlation between the BSA shape vector and BSA, whereas EF correlated less with its EF shape vector. Overall, these results imply that shape features shown by the respective shape modes accounted well for differences in both BSA and EF in our shape population. In a strong correlation between functional parameter and shape vector, all subjects with low EF values would show those shape features given by the EF shape mode for low shape vector values, and vice versa for all subjects with high EF values. Nevertheless, those trends visually confirmed that our method was able to correctly extract 3D shape features from a population of shapes, which are potentially associated with a functional parameter of clinical relevance (Fig. 12). Therefore, the presented method can be used as a research tool to explore a population of 3D shapes, in order to detect where crucial shape changes occur and whether specific geometric parameters are likely to be of functional relevance.

### Limitations and future work

The biggest limitation of our study is the small sample size of 20 subjects, with rather inhomogeneous characteristics in terms of age (range 11.1 to 20.1 years), age at arch intervention (4 days to 5 years after birth) and type of surgery [24]. Thus, results presented in this work are primarily meant to demonstrate the potential of the proposed statistical shape modelling method by studying the association of complex 3D shape features with external, functional parameters such as EF. This could improve the derivation of novel shape biomarkers in future studies. In CoA patients, our method applied to a larger cohort of patients could help answer whether specific arch morphologies such as the gothic arch shape are associated with hypertension post-aortic coarctation repair [15, 37].

## Conclusions

In this paper, we presented a non-parametric shape analysis method based on CMR data from the user-perspective and applied it to a population of aortic arch shapes of patients post-aortic coarctation repair. The process was described in detail in order to make it more accessible to researchers from both clinical and engineering background. The method has the potential of discovering previously unknown shape biomarkers from medical image databases and could thus provide novel insight into the relation between shape and function. Application to larger cohorts could contribute to a better understanding of complex structural disease, improving diagnosis and risk stratification, and could ultimately assist in the development of new surgical approaches.

## Abbreviations

2D, two-dimension(al); 3D, three-dimension(al); BSA, body surface area [m^2^]; CHD, congenital heart disease; CMR, cardiovascular magnetic resonance; CoA, coarctation of the aorta; EDV, end-diastolic volume [ml]; EF, ejection fraction [%]; ICP, iterative closest point algorithm; PDM, point distribution model; SSM, statistical shape model (ling); VMTK, the vascular modelling toolkit.

## Figures and Tables

**Fig. 13:**
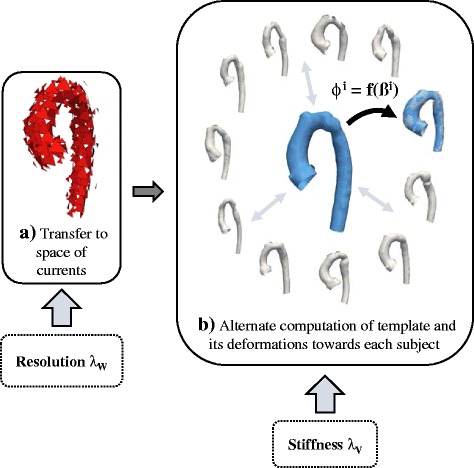
Overview of the template computation using currents. All surface shape models are transferred into their currents representation (**a**). The user has to set the resolution parameter λ_W_ to determine which shape features are to be captured. The template is then computed as the mean shape using an alternate algorithm, minimising the distances between template and each subject (**b**). Thereby, the template shape is initialised as the mean of the currents and then matched with each subject shape. Crucial is the deformation function φ^i^, which is defined by the moment vectors ß that drive the subject-specific deformation of the template. The user has to set the stiffness of those deformations, λ_V_. User input is marked with dashed lines

## Appendix 1: Detailed description of the statistical shape modelling framework

### Forward approach

The shape modelling framework is based on the forward approach [14], which starts from an initial average template shape $\overline{T}$, that is deformed into each subject shape T^i^ by applying an appropriate transformation function φ^i^ (Eq. 5). The subject-specific transformation function φ^i^ deforms the template towards each individual subject shape and thus contains most of the shape information. The residuals ε^i^ correspond to irrelevant shape features such as image artefacts [12].

5 $$ {T}^i={\varphi}^i\cdot \overline{T}+{\varepsilon}^i $$

*“Subject = Deformed Template + Residuals”*

### Mathematical currents as non-parametric shape descriptors

The current of a surface S is defined as the *flux of a test vector field* across that surface. The resulting shape T (a surrogate representation of S) is uniquely characterised by the variations of the flux as the test vector field varies. The definition of currents related to a flux actually stems from Faraday’s law in electromagnetism, where a varying magnetic field induces a current in a wire [20, 38].

### Input parameters: resolution λ_W_ and stiffness λ_V_

Using mathematical currents allows modelling shape as a distribution of specific shape features. Shapes or surface models of shapes (given as computational meshes) are transferred into a vector space W generated by a Gaussian kernel, called the *space of currents* (Fig. 13a) [9]. Similar to histograms, kernels indicate how likely a certain parameter value occurs within a population, i.e. how shape features are distributed. The crucial parameter is the standard deviation of the kernel λ_W_, which allows to control how coarsely or finely a surface is resolved by currents [14]. If λ_W_ is chosen too small, too many shape features are captured and noise dominates, whereas if λ_W_ is chosen too large, important characteristics may be lost [12]. Thus, the parameter λ_W_ is referred to as the *resolution* of the currents representation. It is defined in millimetres and is one of the parameters to be set by the user.

To encode all 3D shape information present in the dataset, the template is transformed, i.e. deformed into each of the shapes used to compute it via transformation functions φ^i^ (Eq. 5). Similar to the definition of the space of currents, subject-specific transformation functions φ^i^ are defined within another vector space V as a Gaussian kernel with standard deviation λ_V_. The parameter λ_V_ controls the *stiffness* of the transformations φ^i^. Intuitively, λ_V_ affects the size of the region that is consistently deformed – the larger λ_V_, the more global (“stiffer”) the deformation; the smaller λ_V_, the more local (“less stiff”) the deformation [12]. λ_V_ is also defined in millimetres and is the second parameter to be set by the user.

### Computing the template in the space of currents, based on transformations

After defining all the shapes present in the population in the space of currents, the template is initially computed as the empirical mean shape $\overline{T}$. To be able to deform the template towards the shape of each individual patient, a suitable transformation function φ^i^ is required. Here, φ^i^ is defined using the large deformation diffeomorphic metric mapping (LDDMM) approach [39]. The transformation φ^i^ is a function of moment vectors ß, which contain the initial kinetic energy necessary to cover the path of a transformation φ^i^ (i.e. deformation) from one current to the other [21]. Moments ß thus “drive” the transformation.

The template $\overline{T}$ and the associated transformations φ^i^ (Eq. 5) are computed simultaneously using an alternate two-step minimisation algorithm (Fig. 13b) [14]. The aim is to minimise the distance in the space of currents between the deformed template $\overline{T}$ and its respective target shape object T^i^. Once the initial template is computed as the empirical mean shape, the distance between template and target is first minimised with respect to the transformations φ^i^, registering the initial template to each target shape independently (“first step”) [14]. The new, updated template is then calculated based on those transformations and the initial template, thus minimising the equation with respect to the template (“second step”). The second step reduces the overall registration error and yields a template that is more centred with respect to the target shapes. This process is iterated until convergence [21]. The template and its deformations towards each individual shape constitute the SSM.

Note that both template $\overline{T}$ and transformations φ^i^ are calculated based on currents i.e. based on surrogate representations of surfaces, not on the actual computational meshes. Therefore, results from the space of currents have to be mapped back to the original space of the computational meshes. The mesh surface model of the final template is obtained by deforming the mesh of the patient closest to the template towards the template currents representation.

### Analysing the output – the concept of shape vectors describing the entire 3D shape

As each patient shape is associated with a large number of moment vectors, output data is difficult to analyse and interpret. Therefore, analysis of shape variability requires a dimensionality reduction in order to discard any redundant shape information while retaining principal contributors to shape variability [12], which can be achieved by applying partial least square regression (PLS) [12, 22]. PLS combines dimensionality reduction in the fashion of a principal component analysis (PCA) [5, 25], with linear regression. Given two sets of variables X (predictors) and Y (response), PLS computes the shape modes which best explain the variance of X, Y and the covariance of X and Y.

As predictors X, the characteristic moment vectors β that parameterise the deformations of the template towards each patient were used. Analysed response variables Y were body surface area (BSA) and the functional parameter ejection fraction (EF). The resulting shape modes were ordered according to their correlation with the response variable Y, with the first one being most correlated and accounting for a certain percentage of variability in the response Y and in the predictors X, which encode all shape information. Here, we retained the first PLS shape mode only in order to capture only shape features *most related* to either BSA or EF and thus to avoid overfitting.

The shape modes describe the main components of shape information present in the population, so that each subject shape in the dataset can be approximated by linearly combining shape modes. Thus, the shape of subject i is characterised by a unique linear combination of m weights for m shape modes as exemplified in (Eq. 6).

6 $$ Shap{e}^i={\displaystyle \sum_{k=1}^m weigh{t}_k^i}\cdot Mod{e}_k $$

Those subject-specific weights constitute the *shape vector* X_S_, which is obtained by projecting each patient transformation onto the shape modes [12]. Correlating the shape vector and response parameter Y shows how well each subject’s shape features (supposedly related to Y) are represented by the derived shape mode.

Mathematical details of how our framework allows deriving PLS shape modes and shape vectors based on deformations can be found in [21] and [12]. PLS shape modes were computed using the *plsregress* function in Matlab.

## Appendix 2: Preliminary sensitivity analysis of meshing and λ parameters

A sensitivity analysis was performed to investigate how the mesh resolution of the input surface models and the setting of the λ parameters affect the template shape and the computational time needed to compute a template. Segmented 3D surface models of 5 randomly chosen aortic arches were meshed from high (5 cells/mm^2^), to medium (2.5 cells/mm^2^) to low (0.5 cells/mm^2^) mesh resolution using VMTK. A template was calculated for each of the differently meshed test sets, first with λ_W_ and λ_V_ being constant at 15mm. All computations were performed on a workstation with 32GB memory using 10 cores. Computational time was recorded. Results showed that the computed template shape was not substantially affected by changing the mesh resolution, whereas computational time increased dramatically for high input mesh resolutions. Using the low-resolution meshes, templates were computed changing λ_W_ from 10 to 15 and 20mm, and λ_V_ from 10 to 20 and 30mm respectively, to investigate the effect of the λ parameters on the template shape. Lower λ_W_ values, i.e. higher resolution increased the computational time needed for the template calculation. The final template shape was substantially influenced by changing both λ_W_ and λ_V_ (Table 5).

**Table 5:** Influence of mesh resolution and shape model parameters λ_W_ and λ_V_ on computational time and template shape

Parameter	Runtime	Template Shape
Mesh Resolution ↑	↑	-
Currents Resolution λ_W_ ↑	↓	↑ ↓
Deformation Stiffness λ_V_ ↑	↑ ↓	↑ ↓

↑ denotes an increase; ↓ denotes a decrease of the parameter; ↑ ↓ denotes an unpredictable change

## Additional files

Additional file 1: Video 1. Computed template (rotating). 360° rotating view of the computed CoA template. (AVI 23282 kb) Additional file 2: Video 2. BSA Shape Mode deformations of the template. Side view of the derived BSA Shape Mode deforming the template; thereby showing shape patterns associated with low and high BSA, respectively. (AVI 3968 kb) Additional file 3: Video 3. EF Shape Mode deformations of the template. Side view of the derived EF Shape Mode deforming the template; thereby showing shape patterns associated with low and high EF, respectively. (AVI 5021 kb)
